# Urogenital Schistosomiasis among Schoolchildren and the Associated Risk Factors in Selected Rural Communities of Kwara State, Nigeria

**DOI:** 10.1155/2018/6913918

**Published:** 2018-05-02

**Authors:** Babamale Olarewaju Abdulkareem, Kolawole Olasunkanmi Habeeb, Abdulganiyu Kazeem, Abdulkareem Olaitan Adam, Ugbomoiko Uade Samuel

**Affiliations:** Department of Zoology, University of Ilorin, Ilorin, Nigeria

## Abstract

Urogenital schistosomiasis is a chronic parasitic disease that causes severe morbidity among schoolchildren in many poor-resource communities in Nigeria. We investigated the prevalence, intensity, and risk factors of the infection in three communities of Kwara State to ascertain the current status of the disease. Of the 724 urine samples screened, using filtration method, 332 (45.6%) school-aged children were infected with average intensity and mean population eggs load of 127.9 eggs/10 ml of urine and 0.794, respectively. Prevalence and intensity of infection varied with communities: high in Ajase-Ipo (57.1%; *X* = 100.7 ± 23.01 eggs/10 ml) and low in Shonga (37.5%; *X* = 91.4 ± 78.0). Infection was significantly (*P* < 0.05) higher in males (50.8%) than the females (42.4%). Similarly, infection significantly (*P* < 0.05) increased with increasing age. Multivariate logistic analysis of risk factors revealed that lack of portable drinking water (adjusted odd ratio (aOR) = 4.76; 95% CI = 2.64–5.98), unemployment (aOR = 2.23; 1.87–2.294), lack of knowledge of infection (aOR = 2.16; 0.59–3.83), and frequent contact with contaminated water bodies (aOR = 2.01; 1.45–2.70) were important predictors of urinary schistosomiasis. Therefore, continuous evaluation of the intervention strategies that address risk factors must compliment Mass Drug Administration to curtail the transmission and debilitating health consequences of infection in endemic settings.

## 1. Introduction

Urogenital schistosomiasis is a common neglected tropical disease in many rural communities in African countries, with patches of infection in the Eastern Mediterranean Region [1–3]. Globally, an estimated 239 million people are currently infected, with burden estimated at more than 3.5 million disability-adjusted life years (DALYs) [4, 5]. In many endemic areas, severely infected individuals may suffer fibrosis of the bladder, kidney damage, bladder cancer, and death if untreated [3]. This, however, depends on several factors such as host-parasite genetics, degree and length of exposure, intensity of infection, host immune response to the parasites, and coinfections with other tropical diseases such as malaria and HIV-1 [6].

In Nigeria, urinary schistosomiasis is a serious health problem with about 29 million infected cases and 101 million people at risk of infection [7–9]. Recent reports showed unabated increase of infection in all the geographical zones of the country, particularly among the schoolchildren [10–12]. Epidemiological studies in many endemic communities have attributed sustained infection to many factors including routine agricultural practices, human behavior, and failed water projects to meet the needs of people [9].

Currently, information on the status of infection in Kwara State is still scanty and the epidemiological knowledge is rudimentary; the baseline data for control interventions is nonexistent. Therefore, this survey investigates the prevalence, intensity, and risk factors of urinary schistosomiasis among the schoolchildren in three rural communities of Kwara State, Nigeria.

## 2. Materials and Methods

### 2.1. Study Areas

The study was conducted in three selected rural communities of Kwara State, namely, Ajase-Ipo (located on Lat. 8° 13′ 60 N and Long. 4° 49′ 0 E), Bacita (Lat. 9° 4′ 59.99′′N and Long. 4° 57′ 0.0′′E), and Shonga (Long. 9° 1′0′′N and Lat. 5° 9′ 0 E) between March 2015 and July 2016. The ecological, behavioral, and population characteristics of these communities are similar, and the climate is tropical, with distinct dry (November–March) and rainy (April–October) seasons. Maximum temperature ranges between 25.7 and 33.7°C. The indigenous people of our study areas, majorly Nupe and Yoruba ethnic groups, are predominantly petty traders and subsistence farmers. Basic infrastructural facilities such as water supply, electricity, and health care services are grossly inadequate, and school enrolment is low with considerable number of school dropout. Environmental and personal hygiene are precarious as human wastes are indiscriminately dumped around human habitation, and animals roam freely in streets. Inhabitants largely depend on contaminated open dug wells, pristine stream, and rivers for domestic water supply.

## 3. Ethical Consideration

The study protocol was approved by the University of Ilorin Research and Ethical Committee (Ref. number UERC/ASN/2014/011). The traditional leaders and an ad hoc ethical committee of Local-Government Authorities gave approval before the commencement of the study. Finally, informed written consent was obtained from each adult subject and the parents/caregivers of each child before enrolment in the study.

## 4. Study Sample

Primary and secondary schoolchildren, aged 4–18 years, were randomly recruited for this study in all the study areas. Menstruating females and individual who had stayed less than 6 months in the communities were exempted from study.

## 5. Urine Sample and Data Collection

Randomly recruited volunteers in each community were given prelabelled clean plastic screw capped bottles to be fully or partially filled with their urine samples between 11.00 and 15.00 hours. At the point of collection of sample questionnaire was given to obtain information on biodata (age, sex, occupation of parents, and duration of stay in community), source of water supply, frequency of water contact, and other risk-related factors of schistosomiasis. Thereafter, all collected urine samples were transported to the Parasitology Laboratory, Department of Zoology of University of Ilorin, for parasitological analysis. Infected individuals were immediately recommended for treatment after another visit to the study areas and laboratory processing of the urine samples were done.

## 6. Sample Processing

All urine samples were collected and preserved in each community and transported to the parasitology laboratory of Department of Zoology, University of Ilorin. Microscopic examination of each urine sample for detection of* S. haematobium* eggs was performed using filtration method. In brief, 10 ml of each urine sample was drawn into a syringe and passed through a Millipore filter (12 *μ*m polycarbonate filter) to recover the eggs. The filter was carefully examined and eggs counted at ×10 objective of a light microscope.

## 7. Data Analysis

All data were double entered using SPSS version 16.0 for Windows (SPSS Inc., Chicago, IL, USA). Differences in proportions were tested by chi-square tests, either for trend or for independence, as appropriate. The mean population eggs load were evaluated and transformed to log_10_⁡(*x* + 1) values to normalize the distribution for statistical analyses. Differences between means were tested using independent samples *t*-test and one-way ANOVA. Logistic regression analysis was performed, using backward elimination, to assess the independent associations between prevalence of infection and selected risk factors.

## 8. Result

A total of 724 participants were enrolled in this study, with 50.8% male and 42.4% female. The average age group was 11.3 years. Overall prevalence and mean intensity of infection were 45.6% and 127.9 eggs/10 ml of urine, respectively. The mean population egg load was high in Ajase-Ipo (1.274) and low in Shonga (1.060) (Table 1). Prevalence of infection was age- and sex-dependent in all the communities except in Shonga. Age group 11–14 years had a significant higher prevalence (*P* < 0.05) in the communities, but prevalence with sex was consistently higher in males than females (Table 2). Intensity of infection paralleled the prevalence rates with significant higher intensity in males than females (325.7 versus 139.6 eggs/10 ml of urine) except in Ajase-Ipo and Shonga communities where intensity of infection was slight (Table 3).

The results of the univariate analysis of risk factors for the infection are presented in Table 4. Frequency of water contact, source of water supply, unemployment, and lack of knowledge of infection were the major significant risk factors observed in the study. In the multivariate logistic regression analysis (Table 5) source of water supply (adjusted odd ration (aOR) of 4.76; 95% CI = 2.64–5.98) and unawareness of infection (aOR = 2.16; 95% CI = 0.59–3.83) were significantly associated with infection.

## 9. Discussion

This study presents one of the few baseline epidemiological data of urogenital schistosomiasis in schoolchildren in resource-poor communities in Kwara State, Nigeria. Our results indicate that urogenital schistosomiasis is a common parasitic disease and major cause of morbidity among Nigerian children and other tropical countries [13–15]. The overall prevalence of 45.6% in our study area is threefold higher than the national Nigerian average of 13% [16], and the mean intensity of 127.9 eggs/10 ml suggests a long-term transmission, which underscores the public health importance of this chronic disease in Kwara State, Nigeria. These findings are comparable to several reports from many endemic regions in Nigeria [17–21] and other endemic countries such as Senegal, Malawi, Madagascar, Tanzania, and Cameroon [22–25]. However, the intensity and prevalence of infection in the current study is slightly lower than the observation in children from other regions within and outside Nigeria [26]. This variation in the occurrence of infection may be ascribed to difference in epidemiological profile. Besides, a single day urine sample used for this study would likely underestimate the severity of infection in the selected communities.

Generally, high prevalence rate of urogenital schistosomiasis in this study may be attributed to the intense water-contact activities observed in our study areas, particularly in Bacita and Shonga. Children were often found playing, swimming, washing their cloth/kitchen utensils, and doing other domestic activities in the river in all the three study areas. Also, majority (52.9%) of our study participants had no knowledge about the transmission of* S. haematobium*; let alone any intervention strategy. This poor awareness about schistosomiasis in this part of Nigeria is similar to observations previously reported in Malawi [27], Zimbabwe [28], and Western Kenya [29]. Therefore, the recent call for global effort to eliminate human schistosomiasis by 2025 by World Health Organization [30] should include health education in schistosomiasis endemic communities of the world.

In the current study, the prevalence and intensity of infection varied widely between communities, with the lowest prevalence in Shonga community. This agrees with previous observations in Nigeria and other areas and is most likely due to differences in the force of transmission of the disease [26, 31, 32]. For instance, the inhabitants of Shonga communities are predominantly peasant farmers and fishermen but provided with borehole water as alternative source of water supply. This may have reduced the water-contact activities of the people when compared with the inhabitants of Bacita that depend solely on river and stream waters for their daily domestic activities. Besides, uneven distribution of infrastructural amenities such as health, toilet, and portable water facilities in our study area may also account for variation in the occurrence of infection in the communities.

Our finding on age and sex pattern of infection is identical to what is obtainable in many endemic communities in Africa [6, 14, 33–35]. The highest prevalence (49.4%) and intensity (147.4 eggs/10 ml of urine) were recorded within the 11–15-year age group. These age groups have been tremendously characterized with boisterous water-related activities such as fishing, washing, swimming, and playing that increase the risk of infection in the communities. This probably accounts for high infection rate among the groups. Similarly, the high infection in male children than the female suggests the higher exposure risk activities in males than the females. This observation indicates that the sex and other factors are important transmission foci in our study communities, and it corroborates findings from many other endemic settings [14, 19, 26, 34–39].

Our analysis of risk factors revealed a significant relationship between prevalence of infection and many factors such as lack of knowledge of infection, occupation, and source of water supply. This conforms to several reports in different ecological regions of Nigeria and sub-Saharan Africa [6, 14, 20, 22, 24, 36–38]. Unemployment status of the parent was observed as a significant predictor of schistosomiasis, and it remained an important risk factor in the multivariate logistic regression analysis (aOR = 2.23). This high rate of unemployment in the study area is a direct indicator of low income and poverty which have been significantly associated with urogenital schistosomiasis.

In our study, only 27% of the study population had a fair knowledge of the cause and prevention of the disease. Many infected male children considered haematuria (blood with urine) as a sign of maturity rather than a symptom of infection. This has serious implication on the control and treatment of infection. Similarly about 63% of the population had no access to pipe-borne water. This possibly influences the high frequency and duration of water contact in the locality.

These findings had demonstrated that urogenital schistosomiasis is endemic and constitute severe morbidity in school children in Kwara State. Therefore, continuous disease evaluation and implementation of a broad-based public health and socioeconomic development that includes provision of clean and safe drinking water and health education are essential to prevent the transmission of infection in the endemic areas.

In conclusion, the recent mass administration of Praziquantel to schoolchildren in the state should be complemented with provision of portable water and strict environmental management in the construction of road, damming and irrigational farming. These will facilitate schistosomiasis elimination in the year 2025 as contained in the global strategic plan.

## Figures and Tables

**Table 1:** Overall prevalence and intensity of urinary schistosomiasis in the selected study communities.

Locations	Number examined	Number infected	Percentage infected (%)	Mean intensity ± std. deviation	Mean population egg load transformed into log^(*x* + 1)^
Overall	724	332	45.6	127.9 ± 67.13	0.794
Bacita	205	106	51.7	130.5 ± 12.34	1.239
Ajase-Ipo	212	121	57.1	100.7 ± 23.01	1.274
Shonga	307	115	37.5	91.4 ± 78.00	1.060
*P* values			**0.014**	**0.018**	**0.039**

**Table 2:** Infection pattern with respect to the age and sex of the study participants.

Locations	Bacita (%)	Ajase-Ipo (%)	Shonga (%)	Total (%)
*Age group*				
≤5	5/2 (40.0)	6/2 (33.3)	0/0 (0.0)	11/4 (36.4)
6–10	85/44 (51.8)	5/3 (60.0)	28/9 (32.1)	118/56 (47.5)
11–14	66/37 (56.1)	125/79 (63.2)	157/56 (35.6)	348/172 (49.4)
≥15	49/23 (46.9)	76/37 (48.7)	122/50 (41.0)	247/110 (44.5)
*P* value	**0.001**	**0.031**	**0.045**	**0.001**
*Sex*				
Male	111/67 (60.4)	119/74 (62.2)	185/70 (37.8)	415/211 (50.8)
Female	94/39 (41.5)	93/47 (50.5)	122/45 (36.9)	309/131 (42.4)
*P* value	**0.011**	**0.046**	**0.180**	**0.047**

**Table 3:** Egg counts of the *S. haematobium* in 10 ml of urine of the infected study populations.

Locations	Bacita (mean ± std.)	Ajase-Ipo (mean ± std.)	Shonga (mean ± std.)	Total (mean ± std.)
*Age group*				
≤5	42.7 ± 16.21	64.5 ± 7.81	0.0 ± 0.0	56.9 ± 34.60
6–10	53.5 ± 13.50	50.3 ± 19.42	41.2 ± 10.11	68.5 ± 15.71
11–14	77.7 ± 39.73	61.9 ± 29.25	76.1 ± 55.19	73.8 ± 21.94
≥15	139.5 ± 85.64	309.3 ± 220.27	89.6 ± 345.93	147.4 ± 67.68
*P* value	**0.001**	**<0.001**	**0.091**	**0.008**
*Sex*				
Male	459.6 ± 43.70	265.4 ± 91.45	101.1 ± 34.81	325.7 ± 50.66
Female	212.3 ± 98.51	206.8 ± 23.76	45.8 ± 54.40	139.6 ± 88.73
*P* value	**<0.001**	**0.531**	**0.092**	**0.004**

**Table 4:** Univariate analysis of epidemiological factors for urinary schistosomiasis in the study area.

Factors	Prevalence	Odd ratio (95% CI)	*P* value
*Occupation of the parent*			
Government worker	195/69 (35.4)	1	
Trader	203/85 (41.9)	1.19 (0.87–2.32)	0.062
Unemployed	326/178 (53.1)	2.12 (1.54–2.39)	0.003
*Frequency of water contact *			
Daily	380/175 (46.1)	1.84 (0.54–2.19)	<0.001
Weekly	134/79 (58.9)	2.01 (1.88–2.49)	0.004
Monthly	210/78 (37.1)	1	
*Source of water*			
Pipe borne	98/36 (36.7)	1	
Well	117/52 (44.4)	0.98 (0.72–1.53)	0.061
Stream/river	509/244 (47.9)	2.77 (2.80–3.01)	0.002
*Duration of activities in water*			
Long (>5 minutes)	479/231 (48.2)	0.58 (0.21–0.83)	0.319
Short (≤5 minutes)	245/101 (41.2)	1	
*Awareness of infection *			
Yes	197/53 (26.9)	1	
No	509/279 (52.9)	2.87 (1.33–3.04)	0.004

**Table 5:** Multivariate analysis of epidemiological factors of urinary schistosomiasis among the infected participants.

Factors	Adjusted odd ratio	95% CI	*P* value
Gender (male)	1.98	0.65–2.19	0.007
Age (≥6 years)	1.41	1.09–1.75	0.421
Unemployment	2.23	1.87–2.94	0.036
Frequent water contact	2.01	1.45–2.70	0.047
Lack of portable water	4.76	2.64–5.98	<0.001
Lack of awareness of infection	2.16	0.59–3.83	0.001
